# Edge-Illumination X-Ray Dark-Field Tomography

**DOI:** 10.1103/PhysRevApplied.19.054042

**Published:** 2023-05-11

**Authors:** Adam Doherty, Savvas Savvidis, Carlos Navarrete-León, Mattia F.M. Gerli, Alessandro Olivo, Marco Endrizzi

**Affiliations:** 1 Department of Medical Physics and Biomedical Engineering, University College London, London WC1E 6BT, United Kingdom; 2 UCL Division of Surgery and Interventional Science, Royal Free Hospital, London NW3 2PF, United Kingdom; 3 Stem Cell and Regenerative Medicine Section, Great Ormond Street Institute of Child Health, University College London, London WC1N 1EH, United Kingdom

## Abstract

Dark-field imaging is an x-ray technique used to highlight subpixel, typically micrometer-scale, density fluctuations. It is often used alongside standard attenuation-based and also phase-contrast x-ray imaging, which both see regular use in tomography. We present x-ray dark-field computed tomography (CT) with a laboratory edge-illumination setup. The dark-field contrast is shown to increase linearly with the x-ray path length through the imaged object, a prerequisite for the use of standard tomographic reconstruction approaches. A multimaterial, custom-built phantom is used to show how dark-field contrast CT can complement attenuation contrast CT for the separation of materials based on their microstructure. As an example of a more complex, biological sample, we present a model rat heart. We show, by comparison with attenuation contrast tomography, that dark-field enables the identification of additional structures undetected through the attenuation contrast channel, as well as offering a consistently sharper reconstructed image.

## I Background

The development of computed tomography (CT) and sophisticated image reconstruction algorithms has enabled x-ray tomography to become one of the most widespread imaging methods in general use, including in medical, security, and manufacturing applications [1]. It requires taking a series of x-ray projections of a sample from different viewing angles, and these are then processed by a reconstruction algorithm to form a three-dimensional (3D) representation of the sample volume. This allows the non-destructive visualization of the sample’s inner structure and overcomes the lack of depth information typical of x-ray radiography.

Since the discovery of x rays at the end of the nineteenth century, the basis for image formation in radiography has traditionally been attenuation contrast. High-atomic-number elements and denser material typically exhibit strong attenuation and are thus characterized by strong image contrast. However, x-ray imaging with samples such as soft tissue can often suffer a lack of contrast because the different tissue types are characterized by similar levels of x-ray attenuation, with dual-energy and phase-contrast x-ray tomography being two approaches showing improved soft-tissue contrast [2,3]. These techniques have evolved to become well established in medical imaging applications [4–6].

In recent years, dark-field imaging has seen increasing attention as a complementary source of x-ray contrast to conventional attenuation-based and phase-contrast imaging. Dark-field imaging exploits the ultrasmall-angle x-ray scattering arising from small inhomogeneities within the sample on length scales typically smaller than the system resolution. Strong signals are seen in dark-field images from porous or fibrous samples, such as composite materials [7,8], bone [9] and lung [10,11].

Both x-ray phase-contrast and dark-field imaging require specialized setups to measure the small refraction and scattering effects brought about by the sample. Often these setups rely on synchrotron radiation; however, several approaches have also been developed that allow dark-field imaging in laboratory settings [12–18]. We focus here on edge illumination as it was shown to be reliable and robust under nonideal conditions [19–21]; this approach to dark-field imaging represents the sample in terms of the probability distribution of scattered radiation field as a function of angle.

X-ray dark-field tomography will enable volumetric assessment of scattering media within a sample. The technique has been seen with other setups in laboratory environments [22,23], which has also led to the development of tensor tomography for mapping fiber orientation [24,25]. The edge-illumination system has previously shown phase-contrast tomography [26,27], we report here on dark-field tomography with a laboratory-based edge-illumination system.

### A Edge-illumination x-ray imaging system

**Fig. 1:**
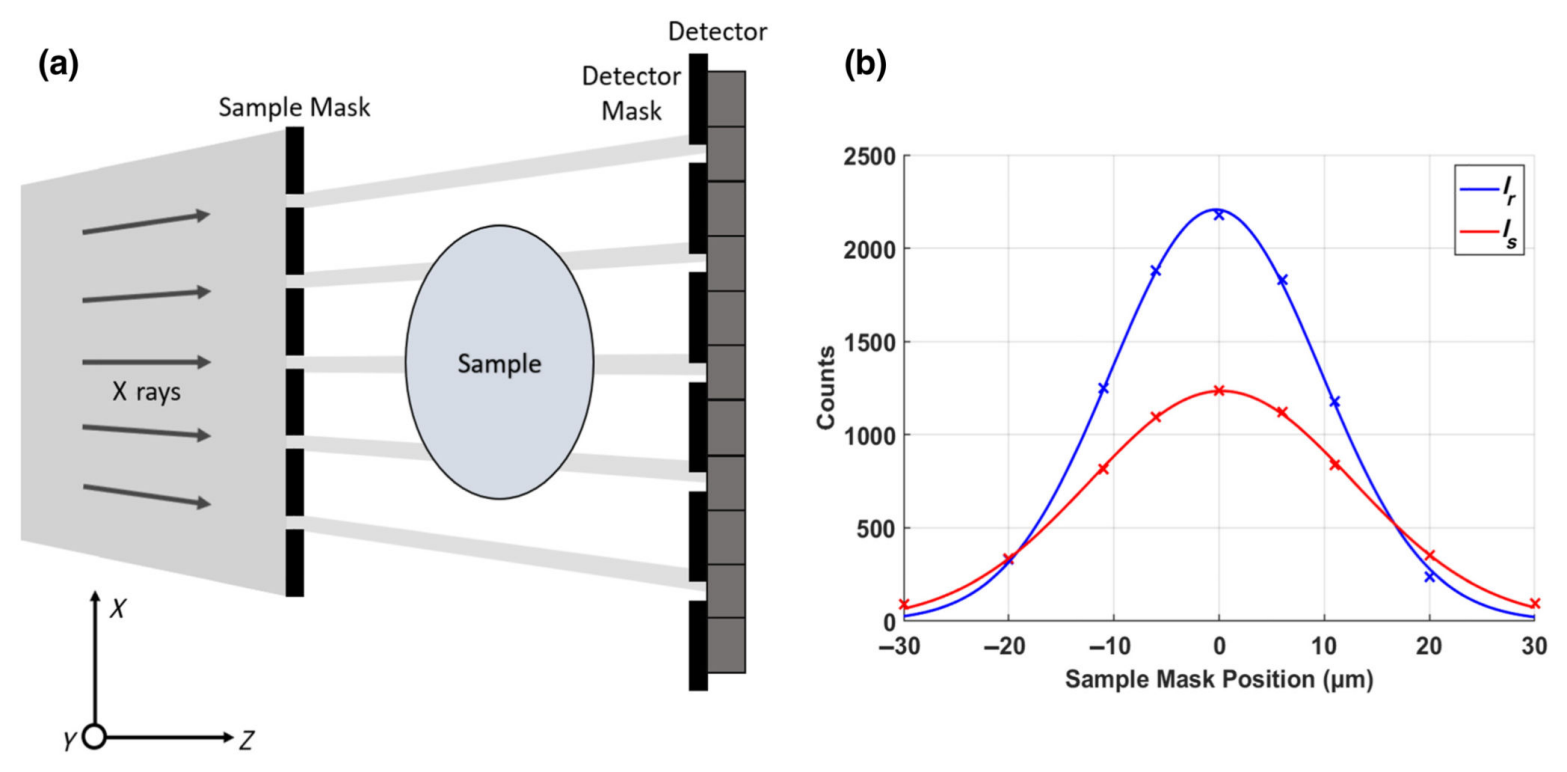
(a) Laboratory edge-illumination setup, consisting of an x-ray beam, sample mask, sample, detector mask and detector. CT rotation is around the *y* axis and dithering is along the *x* axis. An illumination curve scan is carried out by moving the sample mask and sample along the *x* axis through one period (79 μm), which modulates what fraction of the beam reaches the detector through the detector mask apertures. (b) Example illumination curves data points plotted from an acquisition of a cornflour wedge phantom, with the acquired illumination points fit with a Gaussian curve. The blue curve shows the reference illumination curve from the flat-field acquisition, and the red shows an illumination curve within the cornflour sample, with the sample illumination curve reduced in area from attenuation, and broadened due to dark-field scattering.

A typical edge-illumination setup is represented in Fig. 1(a). The system uses a conventional rotating anode source and flat-panel detector, with two absorbing masks used to create sensitivity to angular deflections of x rays from the sample. These masks have apertures arranged periodically and aligned with the system *y* axis, with each mask aperture matched to an aperture of the other mask, and both also matched to a column of pixels on the detector. The experiments outlined in this work used a line-skipped setup [28], where, to avoid pixel crosstalk along the *x* axis, the masks were designed so that the apertures cover every other pixel column.

The system configuration is as described in Havariyoun *et al*. [29]: the sample mask has a period of 79 μm, an aperture size of 10 μm, and is placed 68 cm from the source; the detector mask has a period of 98 μm, an aperture size of 17 μm, and is placed 85 cm from the source. Both masks consist of gold 120 μm thick deposited on a silicon substrate, with a total mask thickness of 400 μm. The source is a molybdenum rotating-anode x-ray source (Rigaku Corporation, Japan) with effective focal spot size 70 μm, which was operated at 40 kV and 20 mA for all experiments shown here. The detector is a CMOS-based flat-panel C9732DK-11 (Hamamatsu, Japan) with a directly deposited 160- μm-thick CsI scintillator and a pixel size of 50 × 50 μm^2^; with line skipping the effective pixel size is increased to 100 × 50 μm^2^.

A typical feature of edge-illumination systems is that the sample is not simultaneously fully illuminated, which requires multiple subsequent exposures with the sample translated in subpixel steps along the *x* axis, a process usually referred to as dithering. Dithering typically increases scan time but should not increase the dose given to the sample as each portion of the sample is still only exposed once per projection, being obscured by the absorbing part of the sample mask for the other dithering steps. In the scans shown here, eight dithering steps were used and stitched together in each projection, reducing the effective pixel dimension along the *x* axis. The effective pixel size at the plane of the sample is 10 × 40 μm^2^.

As the sample mask is moved along the *x* axis and the fraction of the beamlet reaching the detector varies, the illumination on each pixel is modulated through what is known as an illumination curve. Any scattering will deviate x rays away from the center of the illumination curve, but the scattering quantified by dark-field measurements is over a sufficiently small angle that these x rays will be detected towards the tails of the curve. Other scattering phenomena which deviate x rays at higher angles will prevent the photons from reaching the detector and hence contribute to attenuation contrast alongside x-ray absorption. In summary, with a sample in place, the illumination curve will reduce in area from x-ray attenuation, its center will shift along the *x* axis due to x-ray refraction, and it will broaden from ultrasmall-angle x-ray scattering [15].

To separate attenuation, phase, and dark-field signals in an edge-illumination system, at least three intensity projections are required. Each of these projections is acquired with the sample mask at a different relative displacement to the detector and detector masks, which corresponds to taking exposures at different positions on the illumination curve. The process of separating these from the illumination curve is known as phase retrieval, with fitting the illumination curves with Gaussian functions and recovering the three signals reliably and accurately [30]. The edge-illumination system allows for multicontrast imaging, as the attenuation, refraction, and dark-field images are retrieved simultaneously from the same dataset, according to Eq. (3).

## II Methods

Quantifying the dark-field intensity from the ultrasmallangle x-ray scattering signal involves measuring the broadening of the illumination curve with the sample in place. When no sample is present in the setup, the reference illumination curve, *I*_*r*_(*x*), is modeled as a Gaussian function

(1) $$I_{r}(x)=\frac{A}{\sqrt{2\pi \sigma_{r}^{2}}}\exp\left[-\frac{{\left(x-\mu \right)}^{2}}{2\sigma_{r}^{2}}\right]$$

with arbitrary amplitude *A* which accounts for the intensity of the radiation field, and the other two parameters denoting the center, *μ*, and the variance, $\sigma_{r}^{2}$, of the illumination curve. These parameters are defined pixelwise and are the measurements against which the properties of the sample are subsequently measured.

When a sample is placed in the system, the illumination curve changes by the three main mechanisms of attenuation, refraction, and dark-field scattering mentioned previously. The new illumination curve, denoted *I*_*s*_(*x*) for a sample in the x-ray beam, can be described as a convolution between the reference illumination curve above, and the object function, *O(x)*,

(2) $$I_{s}(x)=I_{r}(x)*O(x),$$

where the object function is a single Gaussian whose parameters define the degree of attenuation, refraction, and dark-field scattering from the sample, and * denotes the convolution operation. The full equation for the illumination curve with the sample can now be expressed as

(3) $$I_{s}(x)=\frac{tA}{\sqrt{2\pi \left(\sigma_{r}^{2}+\sigma_{o}^{2}\right)}}\exp\left[-\frac{{\left(x-\mu -\text{Δ}x_{ref}\right)}^{2}}{2\left(\sigma_{r}^{2}+\sigma_{o}^{2}\right)}\right],$$

where *t*, Δ*x*_ref_ and $\sigma_{o}^{2}$, define the area, center, and variance of the object function. Measuring the sample illumination curve and finding the dark-field signal $\sigma_{o}^{2}$ will give the overall broadening of the beamlet as it travels through the sample at a given point in the image plane. However, no depth information is available to deduce where along the x-ray path this broadening occurs, and hence we are unable to recover information on the sample along the *z* axis. To overcome this, the sample can be modeled as a multislice object, with each slice normal to the x-ray propagation along the *z* axis. This model allows the object function to be split into a series of smaller object functions, each originating from a different layer in the sample. If the sample is split into *N* layers, this can be written as a series of convolutions

(4) $$O(x)=O_{1}(x)*O_{2}(x)*\cdots *O_{N}(x)$$

where *O*_*i*_*(x)* is the object function from layer *i*. In this model, the width of the measured object function is simply a summation of the widths from the object functions of each of the individual layers,

(5) $$\sigma_{o}^{2}(x,y)=\sum_{i=1}^{N}\sigma_{i}^{2}(x,y),$$

with $\sigma_{i}^{2}(x,y)$ defining the variance of the object function from layer *i*. By making each layer arbitrarily thick, the sum can be turned into an integral over the path of the x-ray beam. The dark-field signal can now be written as an integral along the path of the x-ray beam through the object at a given CT rotation angle, with this path denoted as *l*, and the rotation angle of the sample denoted by *θ*, which is around the *y* axis in Fig. 1. This integral requires a new parameter, the linear scattering coefficient, denoted by *ϵ*, which describes the width of the scattering function per unit length. Now the measured scattering function can be described at any given projection angle *θ* by

(6) $$\sigma_{o}^{2}(x,y;\theta )=\int \epsilon \left(x^{\prime },y^{\prime },z^{\prime }\right)dl,$$

where *x*′, *y*′, and *z*′ denote the sample coordinates. For tomographic reconstruction, we map this new parameter *ϵ* in a 3D volume, giving each voxel a value for *ϵ*(*x*′, *y*′, *z*′).

The integral of this linear scattering coefficient is analogous to the integral of the linear attenuation coefficient in standard attenuation-based CT. As such, the reconstruction of dark-field tomography volumes can be done using the same algorithms used for regular tomographic reconstructions. One constraint that does apply to dark-field tomography is that the broadening must not extend beyond one voxel, which would invalidate Eq. (6).

As the scattering function is defined as a function of sample mask displacement, the units of the dark-field signal, $\sigma_{o}^{2}$, are typically given in squared length. However, as the dark-field scattering leads to divergence of the beamlet, this broadening will increase with a longer sample-to-detector distance. To remove this dependency, this broadening can instead be quantified through the angular divergence of the beamlet. The result is a dark-field signal with units of squared angle, and a linear scattering coefficient with units of squared angle per unit length.

**Fig. 2:**
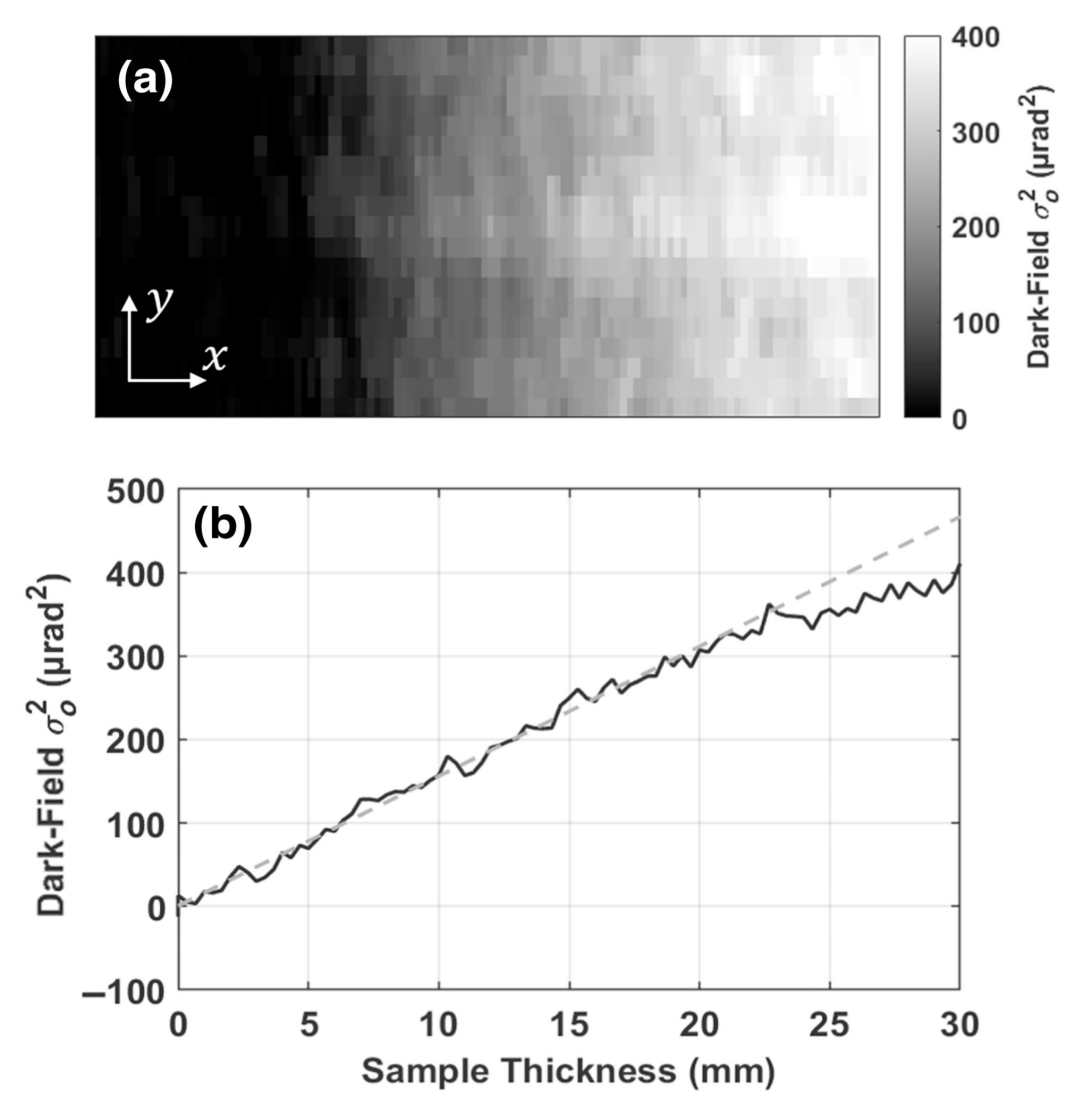
(a) An example of a retrieved dark-field projection for an inhomogeneous wedge phantom, which gets thicker as we move toward the right. (b) A profile of this projection shows the dark-field signal increasing linearly as the thickness of the wedge increases. The measured signal begins to deviate from perfect linear behavior (shown as a dashed line) at approximately 350 μrad^2^, likely due to signal saturation or beam hardening effects.

To test the model and dark-field retrieval up to a chosen scattering strength, a wedge phantom was built of dry cornflour in a triangular sample holder. The thickness increases linearly in one direction of the image projection up to roughly 20 mm, and the dark-field signal would be expected to follow this linearity as per Eq. (6). The planar images were taken with nine illumination curve points across one full period of the sample mask, with exposure times of 1.2 s, and eight dithering steps to sample the whole object. The dark-field image and a linear plot of the intensity profile are shown in Fig. 2.

**Fig. 3:**
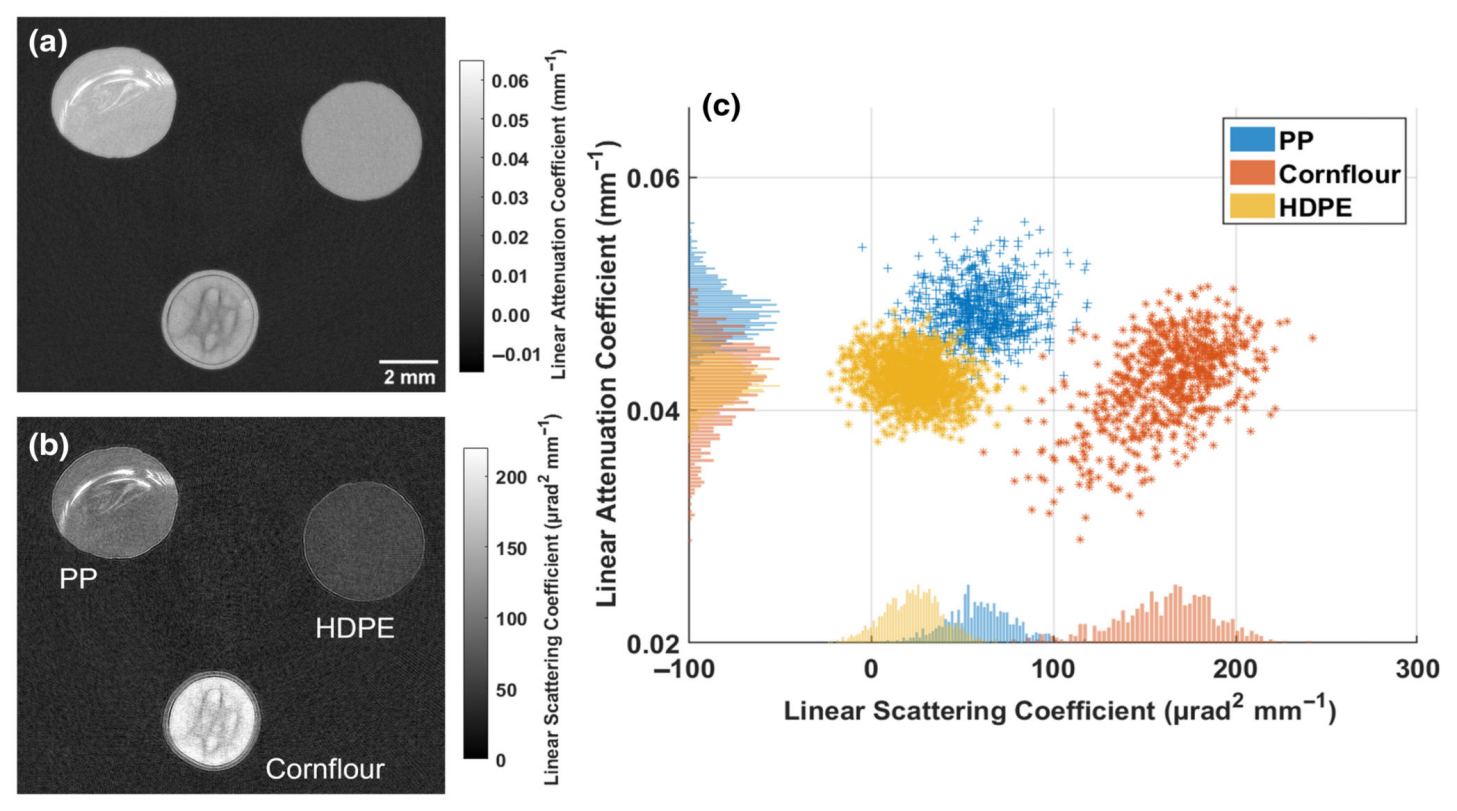
Slices for the phantom for (a) attenuation and (b) dark-field scattering, consisting of high-density polyethylene (HDPE), polypropylene (PP), and dry cornflour. Higher contrast between the three materials can be seen in the dark-field slice. Voxel intensities were binned to form a volume with cubic voxels with dimensions of 0.1 mm. The three materials are distinguishable on a pixel-by-pixel basis, but only when intensities are compared from both images. Note that the intensity from the PP sample was only taken from the uniform lower half of the sample, avoiding the bright defect.

A phantom was then built for a tomographic scan. This was composed of three cylinders of roughly 4 mm in diameter each of different materials: high-density polyethylene (HDPE), polypropylene (PP), and dry cornflour in a plastic straw. This phantom was scanned with 720 projections through 360°, eight dithering steps and seven illumination curve points, resulting in 40 320 projections, each at 1.2 s exposure time. These data were retrieved into attenuation and dark-field projections and reconstructed into two separate volumes. These reconstructions are shown in Fig. 3.

**Fig. 4:**
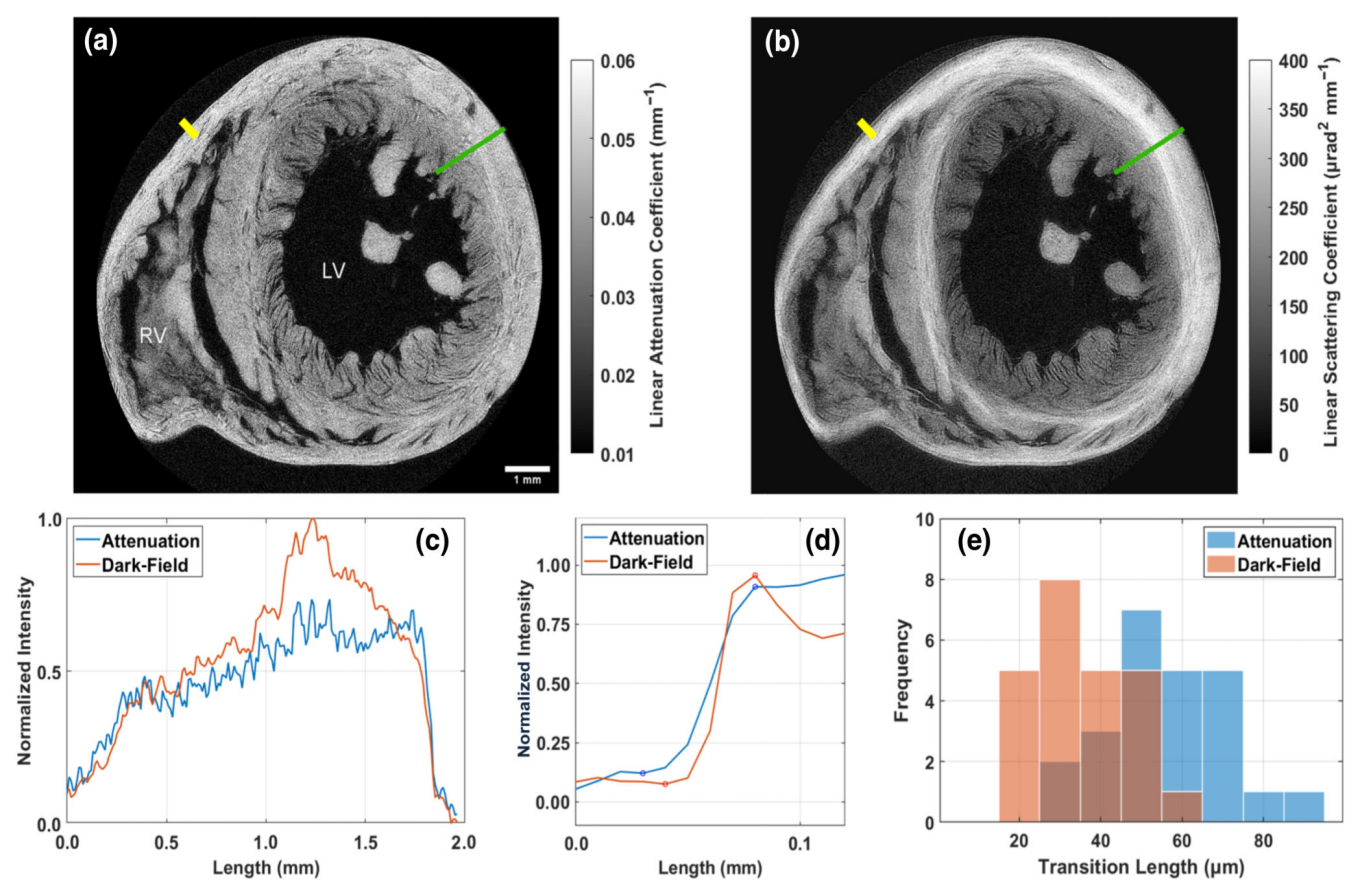
Slices of a rat model heart in (a) attenuation and (b) dark-field channels roughly midway up the ventricular mass, with labeled right and left ventricles (RV and LV). Intensity profiles are seen in (c) from the green line, traveling outwards from the endocardium to the epicardium. The bright layer in the heart wall arises from the difference in fiber orientation moving along this profile, something that does not appear to be visible through the attenuation-contrast channel. The yellow profile was one of 24 tissue-air boundaries measured in quantifying the sharpness of the reconstructions. The average of all these profiles is plotted in (d), with inflection points highlighted to show the shorter transition length in the dark-field reconstruction. The transition lengths for each of the 24 profiles are displayed as a histogram in (e), showing how this is consistently sharper for dark-field tomography (*p <* 3 × 10^−3^).

**Fig. 5:**
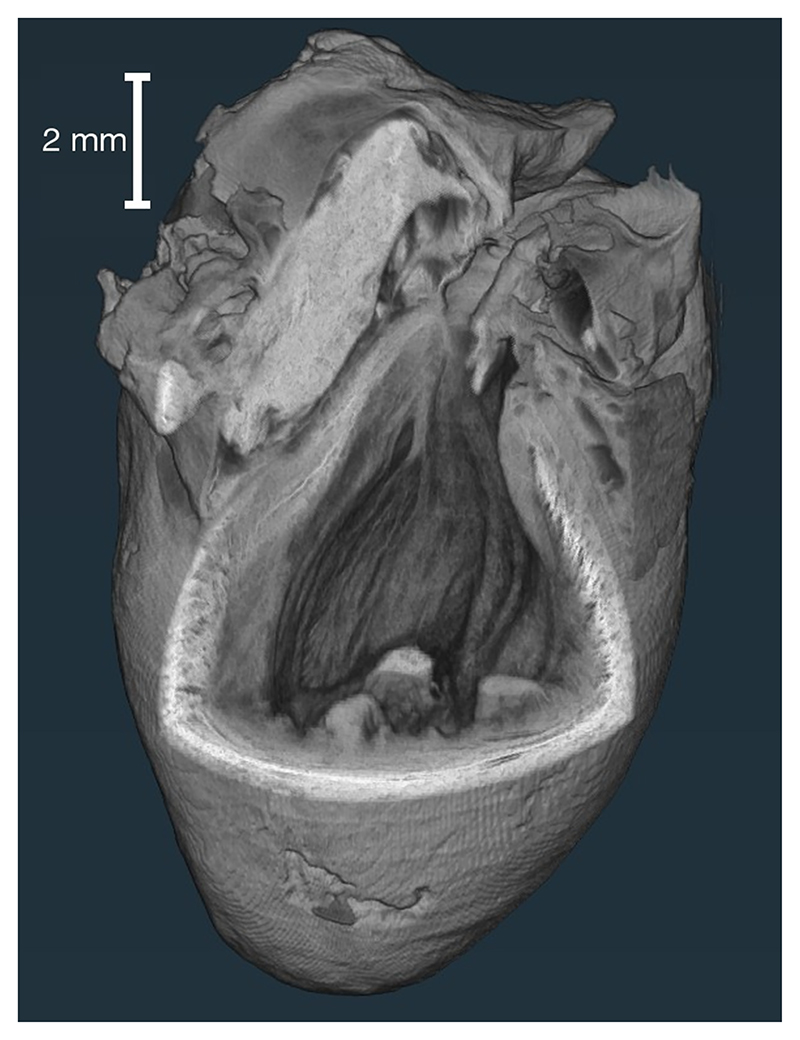
Volume rendering of dark-field signal in the rat heart. The different orientation of fibers across the heart wall appears as the contrast between the inner and outer sections of the wall.

A rat model heart was then imaged to show the application of dark-field tomography in a biological sample. The heart was obtained from the University College London Biological Services Unit, from rats euthanized for organ harvesting via Schedule 1 methods. The specimen underwent fixation (in a 10% paraformaldehyde solution over a 24-h period) and dehydration (in ethanol gradient cycles) before being critically point-dried as per Savvidis *et al*. [31]. The sample was imaged with 1200 projections through 360°, eight dithering steps and seven illumination curve points, with 67 200 projections at 1.2 s of exposure. Reconstructed slices are shown and analyzed in Fig. 4, with a volume rendering of the dark-field dataset shown in Fig. 5.

## III Results and Discussion

The retrieved dark-field projection with the wedge phantom is shown in Fig. 2. The profile shows the dark-field signal increase linearly along the rows of the projection, as the wedge increases in thickness. The signal shows excellent agreement with the expected linear behavior up to approximately 350 μrad^2^, after which some nonlinearity is observed, likely due to signal saturation from strong scatterers. If the dark-field signal measured in projections were to exceed this region, calibrations to compensate for the loss of signal due to partial saturation would need to be considered. Otherwise, other approaches would be required to extend the linear dynamic range of the imaging system, for example by changing the system geometry or mask parameters. Another possible cause of the nonlinearity would be beam hardening [32,33], where changing the source energy might help to extend the linear range. We do not observe values exceeding the linearity region in the other samples presented here, thus we used standard reconstruction algorithms.

The reconstructed slices for the phantom in Fig. 3 show the difference in contrast that is obtained in attenuation and dark-field images. The higher contrast is seen in the dark-field slice, where the three materials are better separated based on their linear scattering coefficient. Voxelwise linear attenuation coefficients are plotted against the corresponding linear scattering coefficient in Fig. 3(c). There is less overlap in the dark-field signal between the three materials and the materials can be best separated based on an analysis that takes into account both images simultaneously. This is linked to the unresolved microstructure of the materials. The differences in density fluctuations are at a length scale which is too small to be visualized by micro-CT at this resolution (100-μm cubic voxels); however, the dark-field image can make these differences visible as they affect the angular spread of the x-ray beam to different extents.

The heart sample showed extra contrast in the dark-field image over the standard attenuation-based slice, highlighted in Fig. 4(c). In both channels, the intensity increases moving outward from the central endocardium wall of the heart towards the outer epicardium wall. This agrees well with the overall increase in fiber density seen in this transition [34]. Additionally, the fibers near the endocardium and epicardium tend to align with the long axis of the heart, parallel to the rotation axis, whereas fibers in the mid-myocardium tend to align circumferentially in the slice plane. The anisotropic scattering from fibrous structures gives dark-field images an extra contrast sensitivity when compared to attenuation, which is independent of fiber orientation around the *z* axis. As such a bright layer is seen in the mid-myocardium in dark-field images. See the Supplemental Material for further analysis of the intensity across the heart wall [35]. This identification of fiber direction in the dark-field imaging is comparable to that seen at a higher resolution with other imaging modalities such as contrast-enhanced magnetic resonance imaging [36] or x-ray phase-contrast imaging at a synchrotron [37].

A further advantage we have observed with dark-field tomography is the improvement in sharpness. The transition region between tissue-air boundaries was measured in 24 locations across a reconstructed slice, with one example highlighted in yellow in Fig. 4(b). The average of these profiles is plotted in Fig. 4(d). For each of these 24 profiles, this transition length was quantified through measurement of trough-to-peak distance, and these measurements are plotted in Fig. 4(e) as histograms. Dark-field tomography provides consistently sharper transition regions, with an average and standard deviation of the transition lengths being 35.4 ± 11.8 μm in the dark-field reconstruction, compared to 56.3 ± 15.0 μm in the attenuation reconstruction. While this might appear to be a modest improvement, it is important to note that it results from a comparison between the attenuation and the dark-field contrast channels where everything else was kept constant. The detector, exposure time, source size, number of photons, and imaging geometry were the same. Therefore this indicates that the observed increase in sharpness is an intrinsic property of dark-field tomography. This improvement in sharpness is in line with increased contrast observed in dark-field radiography, for features that are smaller than the imaging system’s resolution [38].

## IV Conclusion

Dark-field CT has been shown using a laboratory edge-illumination x-ray setup, with a standard data acquisition in line with edge-illumination phase-contrast CT. The dynamic range where the measured dark-field signal was observed to be linear was large enough to allow for 3D imaging of centimeter-sized objects. A custom-built multimaterial phantom was used to show how dark-field contrast can complement standard absorption contrast and enable better material separation. As a demonstration of a more structured biological sample, we showed 3D images of a rat model’s heart. Dark-field CT enabled the identification of the changing fiber structure of the heart wall, something that was invisible in the absorption image. Furthermore, this sample was used to show a consistent sharpness increase across tissue-air boundaries in the dark-field image. We note that this improvement is obtained through the same experimental conditions and appears to be an intrinsic property of dark-field tomography. We believe that the approach presented here, with this enhanced image sharpness and ability to visualize micro-structure changes at length scales that are smaller than the classical system’s spatial resolution, can be a valuable tool for nondestructive inspection of specimens in a laboratory setting.

## Supplementary Material

Supplemental Material
