# Supplementary material for: Edge-Illumination X-Ray Dark-Field Tomography
Source: Phys Rev Appl. Author manuscript; Available in PMC 2025 Apr 15. (PMC7617590; doi:10.1103/PhysRevApplied.19.054042)
Supplement: Supplemental Material [file EMS204037-supplement-Supplemental_Material.pdf]

Supplemental Material: Edge-Illumination X-ray Dark Field Tomography

Adam Doherty,* Savvas Savvidis, Carlos Navarrete-León, Alessandro Olivo, and Marco Endrizzi
Department of Medical Physics and Biomedical Engineering,
University College London, London, WC1E 6BT, UK

Mattia F. M. Gerli
UCL Division of Surgery and Interventional Science, Royal Free Hospital, London, UK and
Stem Cell and Regenerative Medicine Section, Great Ormond Street Institute of Child Health,
University College London, London, WC1N 1EH, UK
(Dated: April 19, 2023)

Further analysis of the heart sample is presented in Fig. 1. Volume renderings for the data show how the contrast between different sections of the heart wall is improved in dark-field imaging. This is due to the orientation of fibres, with the fibres in the bright layer orientated circumferentially around the heart wall, which point out of the image in this cross section. These scatter more than the longitudinally orientated fibres (i.e. moving up and down in this image) that exist towards the interior and exterior of the heart wall.

From the volume rendering, it is clear that this bright central section of the heart wall extends around the whole outer ventricle wall. It is also visible, although less clear, at the top of the left ventricle, in the internal heart wall between the ventricle and atrium. Taking a profile across this internal wall reveals this layer well. In Fig. 1(f), and distinct regions of the heart wall can be separated based on the dark-field signal. This is something that is not present in the attenuation contrast profile, which just shows a decrease in density towards the ventricular side of the wall.

* adam.doherty@ucl.ac.uk

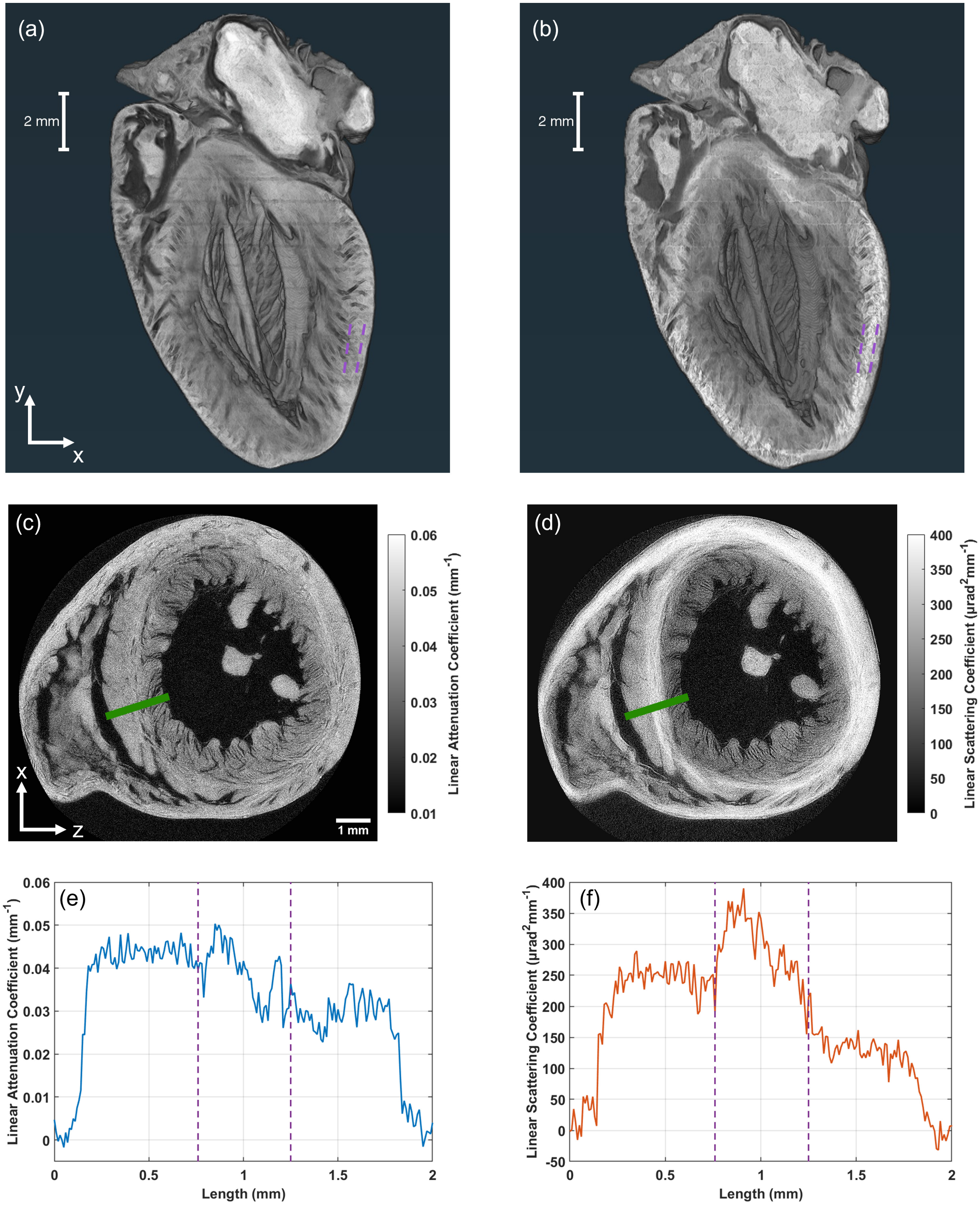

FIG. 1: Volume rendering of rat heart in (a) attenuation and (b) dark-field contrast channels. A bright section in the heart wall is visible in the dark-field rendering but is not visible in attenuation, highlighted by the purple lines separating these sections. The attenuation and dark-field slices are presented in (c) and (d) respectively, with a profile taken across the internal heart wall (green line) and plotted in (e) and (f) for the two contrast channels, going from left to right. In dark-field, this profile shows a large signal towards the centre of the wall, indicating fibres orientated in the slice plane, which is not visible in the attenuation profile.
